# School Climate, Learning Behavior Patterns, and Procrastination: Emotional and Motivational Pathways to School Success

**DOI:** 10.3390/ejihpe16080113

**Published:** 2026-08-01

**Authors:** Luana Sorrenti, Carmelo Francesco Meduri, Concettina Caparello, Pina Filippello

**Affiliations:** 1 Department of Clinical and Experimental Medicine, University of Messina, 98100 Messina, Italy; sorrentil@unime.it (L.S.); gfilippello@unime.it (P.F.); 2 Department of Health Sciences, University Magna Graecia of Catanzaro, 88100 Catanzaro, Italy; 3 Department of Classical, Linguistic and Educational Studies, Kore University of Enna, 94100 Enna, Italy; concettina.caparello@unikore.it

**Keywords:** school success, academic procrastination, school climate, learning behavior patterns, mastery orientation, learned helplessness

## Abstract

The literature has shown that contextual and individual factors of an emotional and motivational nature are often associated with good academic performance and regular school attendance. The present study aimed to examine indirect associations involving Academic Procrastination and School Climate in the relationship between learning behavior patterns (MO-LH) and school success in a sample of 539 Italian secondary school students (M*_age_* = 16.5). Structural equation modeling was used to test the hypothesized relationships. The results showed that LH was positively associated with Academic Procrastination, whereas MO was negatively associated with it. Academic Procrastination was negatively associated with all dimensions of perceived School Climate (Teacher Support, Peer Connectedness, School Connectedness, Affirming Diversity, Rule Clarity, and Reporting and Seeking Help). School Achievement was positively associated with MO, School Connectedness, and Affirming Diversity, and negatively associated with Peer Connectedness. School Absence was negatively associated with MO. Mediation analyses suggested significant indirect associations between learning behavior patterns and school achievement, with Academic Procrastination and Affirming Diversity emerging as potential mediating variables. Findings highlight the importance of addressing emotional and motivational vulnerabilities and fostering a supportive school climate as potential factors associated with adaptive academic functioning and reduced maladaptive outcomes.

## 1. Introduction

### 1.1. Regular Attendance and School Achievement as Indicators of School Success

The primary aim of the education system is to promote the educational and school success of every student by supporting their cognitive, emotional, and social development, so that they can acquire the basic skills necessary for their personal, social, and professional futures. (Fini, 2007; Kearney & Graczyk, 2014).

Different studies use this concept to broadly encompass the various academic outcomes that students can achieve (York et al., 2015). Some institutions consider success to be simply the outcome of student performance, while others place greater emphasis on commitment, participation, and consistency in the learning process (Nunn, 2014).

Currently, school success is considered a complex construct that includes not only indicators of academic performance, such as grade point averages, but also cognitive, relational, and motivational factors that characterize students’ involvement in school life and contribute to their well-being (York et al., 2015; Allen et al., 2017; Gill et al., 2021).

In this context, regular school attendance is key. Consistent participation in lessons facilitates the acquisition of curricular skills and reduces the risk of falling behind and developing learning gaps. Daily participation in educational activities is essential for achieving academic goals, promoting academic success and personal growth, and developing life skills (Nieuwoudt, 2020). For this reason, school attendance is increasingly recognized as an important indicator of the effectiveness of educational programs and the success of school systems (Liu et al., 2021). In this regard, students who attend school regularly tend to achieve better academic outcomes and higher levels of well-being, as consistent attendance facilitates access to learning resources and interactions with teachers and peers (Caparello et al., 2025; Credé et al., 2010; La Rocca et al., 2024). School attendance is a measurable indicator (Gershenson, 2016) that is closely linked to school and personal success, as it shows a consistent correlation with better school performance (Gottfried, 2009; Aucejo & Romano, 2013; Gershenson & Langbein, 2015). Furthermore, among the factors commonly included is the concept of school success; a quantifiable indicator of student performance is the grade point average, which is often used as a measure of school achievement (Venezia et al., 2005).

### 1.2. The Role of Learning Behavior Patterns and Procrastination in School Success

A crucial part of the learning process is how students respond to obstacles (Filippello et al., 2020). Students can adopt adaptive responses, aimed at increasing their sense of competence, or maladaptive responses, which lead them to interpret their school experiences negatively (Sorrenti et al., 2024; Filippello et al., 2020; Diener & Dweck, 1980). While the adaptive response is defined as mastery orientation (MO) and is common to all students who interpret every experience as a learning opportunity, the maladaptive response is defined as Learned Helplessness (LH) and characterizes students who, due to low self-confidence, have low positive expectations. Such a learning behavioral pattern can be conceptualized as a coherent system of learning activities, beliefs, and motivational orientations, reflecting internal patterns that guide students’ responses to academic challenges and encompassing cognitive, motivational, and self-regulatory components (Vermunt & Donche, 2017). In this perspective, MO students, attributing intrinsic value to learning, have greater control over their study process, achieving better results (Sorrenti et al., 2018) and maintaining future expectations of success (Sorrenti et al., 2018; Poredos & Puklek, 2017). Instead, LH students tend to attribute any cause of success in learning to external factors, attributing any educational failures to a lack of ability (Diener & Dweck, 1980; Seligman & Maier, 1967). When faced with complex tasks that require perseverance, these students tend to avoid them or request simpler tasks to avoid failure (Filippello et al., 2020). It is precisely the fear of failure and the belief that one cannot influence one’s own results that often fuel procrastination (Schouwenburg, 1992), which becomes a defense strategy to avoid confrontation with possible failure. Procrastination is a very common irrational behavior which, in the school environment, leads students to voluntarily postpone an educational activity or task even though this delay may have negative consequences (González-Brignardello et al., 2023; Steel, 2007). Although procrastinators would like to complete the activities entrusted to them, they find it difficult precisely because they are afraid of making mistakes or failing (González-Brignardello et al., 2023; Schouwenburg, 1992). Procrastination has also been conceptualized as the result of dysfunctional motivational regulation (Bäulke et al., 2021; Schwinger et al., 2009; Wolters, 2003), which alters the regular learning process of students. Dysfunctional motivational responses, low self-confidence, and a constant fear of failure may lead students to repeatedly postpone their educational activities (Bäulke et al., 2021; Klassen & Kuzucu, 2009; Lee, 2005), negatively impacting academic achievement (K. R. Kim & Seo, 2015). These processes can be coherently interpreted within the framework of social cognitive theory, which posits that students’ academic functioning emerges from the dynamic reciprocal interaction between personal motivational beliefs, behavioral patterns, affective reactions, and self-regulatory processes (Schunk & DiBenedetto, 2023).

### 1.3. Role of School Climate in Daily School Life

There are various definitions of school climate in the literature, but most conceptualize it as the set of students’ perceptions regarding the relational, normative, and value characteristics promoted and shared within the school (Aldridge & Ala’I, 2013; Brookover, 1985). School climate thus refers to the perceived characteristics of the educational environment that shape students’ school experiences, influencing their participation, engagement, and sense of belonging. Given that school climate is conceptualized as a subjective rather than an objective environmental characteristic, students’ behavioral and emotional experiences may also shape how they interpret and evaluate their school context. Indeed, inspired by the main interactionist theoretical approaches, Aldridge and Ala’I (2013) highlight how the school environment can either support or hinder students’ individual educational needs. The authors analyzed the essential components of the educational environment and identified several key dimensions of School Climate. Teacher Support and Peer Connectedness represent relational dimensions, focusing, respectively, on the relationship between teacher and student, and between student and peer group. The dimension of School Connectedness reflects students’ perception of their sense of belonging to, and connection with, the school. Affirming Diversity expresses the school’s commitment to promoting an inclusive environment that values the individual, cultural and linguistic differences of each student. Finally, Rule Clarity and Reporting and Seeking Help concern how clearly school rules are communicated and whether students feel able to report violations or abuse and be listened to and protected within the school environment.

Recent research highlights how a positive school climate can significantly affect student motivation, promoting greater involvement in educational activities and more consistent participation in school life (Lombardi et al., 2019; Sorrenti et al., 2025a). A school environment characterized by trusting relationships, teacher support, a sense of belonging, and an appreciation of diversity contributes to strengthening students’ perceived competence and interest in learning—key features of a mastery-oriented student (Sorrenti et al., 2018). Several studies have also shown that these conditions promote school success, understood as both regular attendance and good academic performance, reducing disengagement and absenteeism (Daily et al., 2020; Durham & Connolly, 2017; Fryer et al., 2018). Conversely, an extremely rigid school environment that pays little attention to interpersonal relationships and is less attentive to inclusion may be less motivating and may lead students to postpone tasks due to a fear of failure, ultimately fostering a sense of helplessness and diminished confidence in their own abilities. These difficulties may, in turn, manifest in procrastination behaviors, along with feelings of helplessness and reduced self-confidence (Rouzbahani et al., 2024; Daily et al., 2020; Sorrenti et al., 2018; Cananoğlu & Tümkaya, 2011). This pattern is consistently associated with a reduced sense of both academic and personal competence and appears to play a significant role in compromising regular school attendance (Daily et al., 2020) and negatively affecting students’ well-being (Sorrenti et al., 2025b).

In line with social cognitive theory, perceived school climate can be understood as a contextual and perceptual construct that interacts with students’ personal beliefs and behaviors (Bandura, 2014; Thapa et al., 2013). While school climate reflects characteristics of the educational context, the present study focuses specifically on students’ perceptions of these characteristics. Therefore, students’ behavioral and emotional experiences may be associated with how they interpret and evaluate their school environment. Accordingly, procrastination may not alter the objective school context but may be related to less favorable perceptions of school climate.

Although previous research has examined the associations between learning behavior patterns, academic procrastination, perceived school climate, and school success, these variables have generally been investigated separately or through isolated relationships. Therefore, drawing on social cognitive theory, the present study aims to address this gap by examining the interrelationships among these variables within an integrated theoretical model.

## 2. The Present Study

In line with previous research (Gershenson, 2016; Aucejo & Romano, 2013; Gottfried, 2009), school attendance and school achievement represent key indicators of school success.

Grounded in socio-cognitive theory (Schunk & DiBenedetto, 2023; Bandura, 2014), which posits that student performance stems from the dynamic interaction between personal, behavioral, and environmental factors, this study examines MO and LH as internal behavioral patterns that influence engagement, emotional responses, and academic performance. In this context, LH—characterized by a fear of failure and low self-efficacy—is associated with maladaptive self-regulation processes, including procrastination as a defensive response to failure-related expectations, and with less positive perceptions of the school climate. Although the school climate is usually considered a contextual factor, students’ perceptions of the school environment may also be related to their motivation and learning experiences. In fact, previous research suggests that individual characteristics and patterns of school functioning are linked to more favorable or less favorable perceptions of the school climate (Molinari & Grazia, 2023; Singla et al., 2021; Koth et al., 2008).

Consequently, LH has been associated with procrastination, motivational dysregulation, and lower academic performance (K. R. Kim & Seo, 2015; Bäulke et al., 2021), whereas, conversely, MO has been associated with rapid engagement in academic demands and a reduced tendency to procrastinate, contributing to a more positive perception of the school climate and ultimately to higher levels of school achievement (Caraway et al., 2003; Lombardi et al., 2019; Sorrenti et al., 2025a).

Even though previous studies have analyzed associations among LH, MO, Academic Procrastination, perceived School Climate, and school success (Sorrenti et al., 2025a; Daily et al., 2020; Aldridge & Ala’I, 2013; Credé et al., 2010; J. Kim & Gentle-Genitty, 2020; Filippello et al., 2020; Diener & Dweck, 1980; Bäulke et al., 2021; Klassen & Kuzucu, 2009; Lee, 2005; K. R. Kim & Seo, 2015), limited attention has been devoted to examining how learning behavior patterns, Academic Procrastination, and perceived School Climate are jointly associated with school outcomes within an integrated socio-cognitive framework. Therefore, consistent with previous research (Duru et al., 2024; Buzzai et al., 2021; Prihadi, 2018; Krihadi et al., 2018), the present cross-sectional study explores the associations among these variables within a theoretically informed framework, without drawing causal inferences. Accordingly, the proposed ordering should be interpreted as reflecting a theoretically derived pattern of associations rather than a definitive causal sequence. For this reason, the primary aim of this study is to examine whether the associations between learning behavior patterns (LH and MO) and indicators of school success (school attendance and academic achievement) are statistically consistent with a theoretically derived indirect pathway involving procrastination and perceived school climate.

Specifically, we hypothesize that LH will be positively associated with procrastination and negatively associated with school climate and school success. Conversely, we hypothesize that MO will be negatively associated with procrastination and positively associated with school climate and school success.

## 3. Methods

### 3.1. Participants

The study sample consisted of 539 students: 304 female (56.4%), 222 male (41.2%), and 13 (2.4%) who preferred not to specify their gender. Participants’ mean age was 16.5 years (SD = 0.99; range = 14–20); most participants were Italian nationals (n = 514), while 25 students were foreign nationals. Students were recruited from secondary schools in southern Italy. The students’ current school average was 7.40 (SD = 0.99, range = 6–10), while the average of school absences, referring to the previous school year, is 23.11 (SD = 11.8, range = 0–80). In Italian schools, the number of absences constitutes a further relevant indicator of educational progress. Generally, up to 25 absences during the school year are considered acceptable and have no consequences on the educational path. Exceeding this threshold can lead to a reduction in school credit, while a number of absences exceeding 50 can carry the risk of not being admitted to the following school year. Students with special educational needs were excluded from the study.

### 3.2. Instruments

A demographic questionnaire was used to collect the participants’ basic information, including age, gender, nationality, educational level, school achievement, school absence, and socioeconomic status (SES).

School Achievement was assessed using students’ self-reported current grade point average, obtained after reviewing their electronic grade records. In the Italian educational system, grades range from 1 to 10 (with 6 = sufficient, 7 = good, 8 = very good, 9 = excellent, and 10 = outstanding). School Absence was measured by asking students to report the total number of days they were absent during the current school year.

Learning behavior patterns were assessed using the Italian version of the Learned Helplessness Questionnaire (LHQ; Sorrenti et al., 2015). The questionnaire consists of 13 items divided into two subscales: Learned Helplessness (LH; 7 items, e.g., “When you encounter an obstacle in schoolwork, you get discouraged and stop trying”) and Mastery Orientation (MO; 6 items, e.g., “I express enthusiasm about my schoolwork”). Participants rated their agreement on a 5-point Likert scale (1 = not true; 5 = absolutely true). Previous studies have demonstrated the reliability and validity of the LHQ (Buzzai et al., 2021; Filippello et al., 2015, 2020).

Academic Procrastination was measured using the Academic Procrastination Scale–Short Form (APS-S; Yockey, 2016). The scale consists of 5 items (e.g., “When given an assignment, I usually put it away and forget about it until it is almost due”), rated on a 5-point Likert-type scale (1 = agree; 5 = disagree). The APS-S has demonstrated good reliability and validity in previous research (Brando-Garrido et al., 2020; Chakraborty & Chechi, 2019; Martín-Puga et al., 2022; Yockey, 2016).

School climate was assessed using the What’s Happening In This School? (WHITS) questionnaire (Aldridge & Ala’I, 2013). The instrument consists of 48 items measuring six dimensions: Teacher Support (8 items; e.g., “At this school teachers take an interest in my background”), Peer Connectedness (8 items; e.g., “At this school I make friends with people from different backgrounds”), School Connectedness (8 items; e.g., “At this school I feel welcome”), Affirming Diversity (8 items; e.g., “At this school my cultural background is respected by students”), Rule Clarity (8 items; e.g., “At this school the rules make it clear that certain behaviors are unacceptable”), and Reporting and Seeking Help (8 items; e.g., “I can report incidents without others finding out”). Responses were provided on a 5-point Likert scale (1 = almost never; 5 = almost always). The original English version of the WHITS was translated into Italian, following the standard translation and back-translation procedure to ensure linguistic and conceptual equivalence. In the present study, a confirmatory factor analysis (CFA) was also conducted (CFI = 0.86; TLI = 0.85; RMSEA (90% CI) = 0.06 [0.06, 0.07]; SRMR = 0.06; *p* < 0.001). In line with recent methodological recommendations (Goretzko et al., 2024), the WHITS demonstrated satisfactory psychometric properties in the present study and showed good internal consistency, consistent with previous research (Aldridge & Ala’I, 2013; Fernández-Sogorb et al., 2026; Sattler et al., 2022).

### 3.3. Procedure

This study was conducted in accordance with the Code of Ethics of the Italian Association of Psychology (AIP). All participants taking part in the research project provided written informed consent, in line with the principles of the Declaration of Helsinki (2013). For minor participants, informed consent was provided by their parents or legal guardians. This study was conducted as part of the international Erasmus+ research project SOS-Attendance and received ethical approval from the University of Alicante’s Ethics Committee (UA-2023-03-07). This article does not report on any animal studies conducted by the authors.

Participants completed the questionnaires in a single session lasting approximately 30–40 min, administered via the Google Forms platform. The anonymity and confidentiality of participants’ responses were strictly guaranteed throughout the entire research process.

### 3.4. Data Analysis

Descriptive statistics, Pearson correlations, and internal consistency (Cronbach’s alpha) were calculated using Jamovi software (Version 2.5.6; The Jamovi Project, 2022). Structural equation modeling (SEM) was conducted in RStudio (Version 2024.04.2; RStudio Team, 2015) using the lavaan package (Rosseel, 2012). SEM was chosen to estimate latent constructs while accounting for measurement errors (Coffman & MacCallum, 2005; Kline, 2023; Iacobucci et al., 2007). To construct latent variables, item parcels were created by aggregating two or more items from the same scale, following procedures described by Little et al. (2007) and Matsunaga (2008). This technique enhances indicator reliability, improves model parsimony, reduces error variance, and facilitates more normally distributed indicators (Bagozzi & Edwards, 1998; Marsh et al., 2004; MacCallum et al., 1999). Consistent with the literature, three parcels were created for each latent construct whenever possible. Using three indicators per latent factor helps to ensure a just-identified measurement model while still providing multiple indicators that reduce estimation bias (Bandalos, 2002). However, for some constructs—Academic Procrastination, School Achievement, and School Absence—item parceling was not possible because these scales included too few items. In these cases, the individual items were used directly as indicators of the latent variables.

Model fit was assessed using several indices: the Comparative Fit Index (CFI), the Root Mean Square Error of Approximation (RMSEA) with its 90% confidence interval, and the Standardized Root Mean Square Residual (SRMR). Model fit was considered acceptable if CFI > 0.90, RMSEA < 0.08, and SRMR < 0.08 (Hair et al., 1998; Kline, 2015). Indirect and direct effects were tested using bootstrapping (5000 samples) with 95% bias-corrected confidence intervals, as recommended by Shrout and Bolger (2002), Preacher and Hayes (2008), and Wu and Jia (2013).

## 4. Results

### 4.1. Descriptive Statistics and Consistency

Descriptive analyses and internal consistency values for all study variables are presented in Table 1. All variables demonstrated acceptable distributional properties, as indicated by skewness and kurtosis values within recommended ranges. All subscales showed adequate internal consistency. Correlational analyses of the variables are presented in Table 2.

### 4.2. Mediation

**Figure 1:**
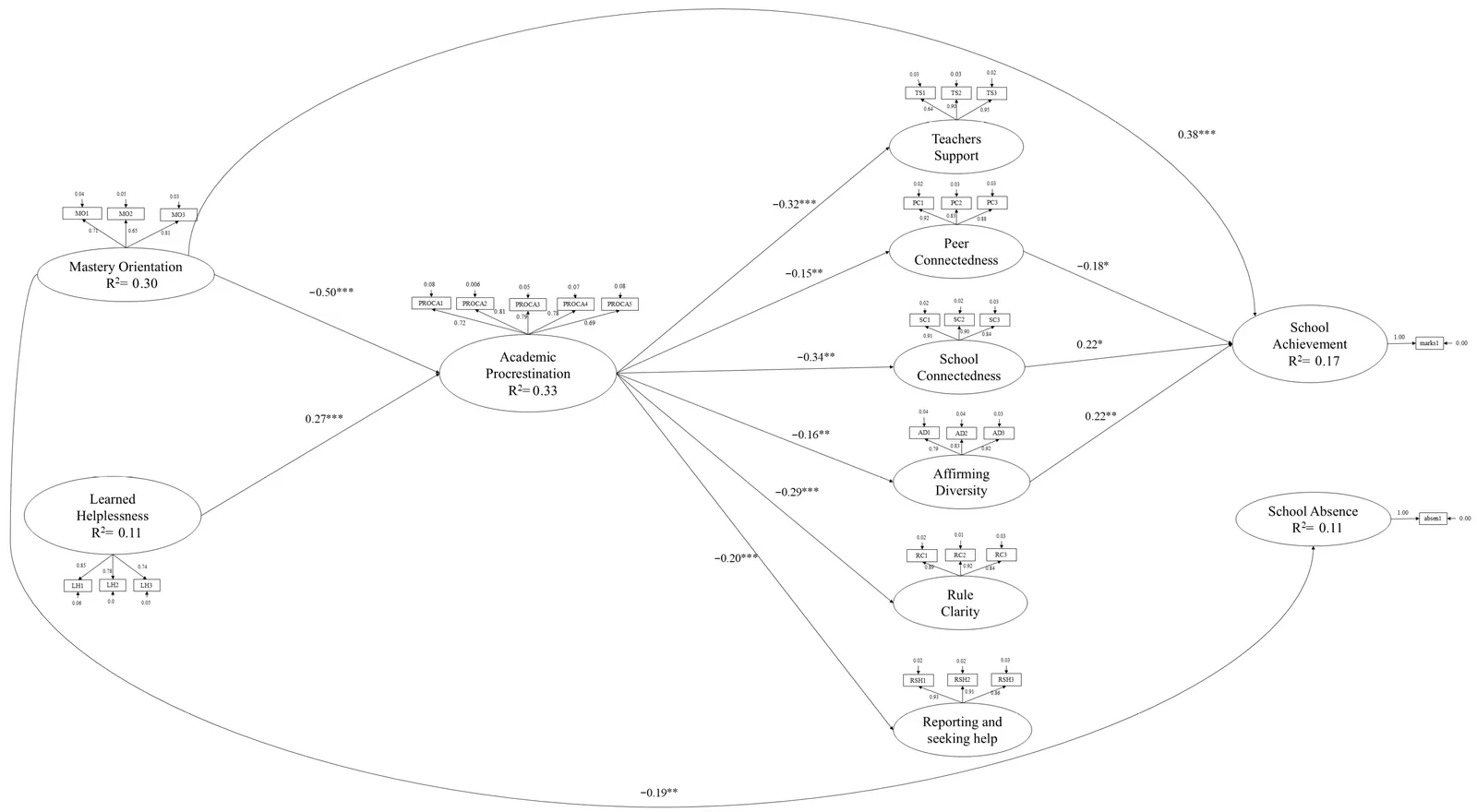
Full mediation model. ***Note:*** *** *p* ≤ 0.001, ** *p* ≤ 0.01, and * *p* ≤ 0.05. Coefficients shown are standardized direct path coefficients. *β* represents standardized coefficients. The insignificant paths have not been inserted. Coefficients’ correlation: Teachers Support <--> Peer Connectedness: 0.40 ***; Teachers Support <--> School Connectedness: 0.59 ***; Teachers Support <--> Affirming Diversity: 0.40 ***; Teachers Support <--> Rule Clarity: 0.48 ***; Teachers Support <--> Reporting and Seeking Help: 0.57 ***; Peer Connectedness <--> School Connectedness: 0.76 ***; Peer Connectedness <--> Affirming Diversity: 0.45 ***; Peer Connectedness <--> Rule Clarity:0.38 ***; Peer Connectedness <--> Reporting and Seeking Help: 0.44 ***; School Connectedness <--> Affirming Diversity: 0.54 ***; School Connectedness <--> Rule Clarity: 0.60 ***; School Connectedness <--> Reporting and Seeking Help: 0.62 ***; Affirming Diversity<--> Rule Clarity:0.61 ***; Affirming Diversity<--> Reporting and Seeking Help: 0.55 ***; Rule Clarity <--> Reporting and Seeking Help: 0.72 **; Learned Helplessness <--> Mastery Orientation: −0.12 *; School Achievement <--> School Absence: −0.27 ***.

Structural equation modeling (SEM) was employed with latent variables to examine the mediating role of Academic Procrastination and students’ perception of the school climate—Teachers Support, Peer Connectedness, School Connectedness, Affirming Diversity, Rule Clarity, and Reporting and Seeking Help—in the relationship between learning behaviors (LH and MO) and both School Achievement and School Absence. The model demonstrated a good fit to the data: χ^2^(395) = 1034.941, *p* = 0.000, CFI = 0.94, SRMR = 0.07, and RMSEA (90%CI) = 0.055(0.051, 0.058). All the indicators loaded significantly on their respective latent variables, with standardized factor loadings ranging from 0.64 to 0.92. All coefficients (β) reported are standardized estimates. Figure 1 illustrates the significant direct paths identified in the structural model.

The results (see Figure 1) showed that Academic Procrastination was positively associated with LH (β = 0.27, *p* ≤ 0.001) and negatively associated with MO (β = −0.50, *p* ≤ 0.001). Regarding students’ perception of the school climate, Academic Procrastination emerged as significantly negatively associated with all School Climate dimensions, such as Teacher Support (β = −0.32, *p* ≤ 0.001), Peer Connectedness (β = −0.15, *p* ≤ 0.01), School Connectedness (β = −0.34, *p* ≤ 0.01), Affirming Diversity (β = −0.16, *p* ≤ 0.01), Rule Clarity (β = −0.29, *p* ≤ 0.001), and Reporting and Seeking Help (β = −0.20, *p* ≤ 0.001). Furthermore, School Achievement was positively associated with MO (β = 0.38, *p* ≤ 0.001), School Connectedness (β = 0.22, *p* ≤ 0.05), and Affirming Diversity (β = 0.22, *p* ≤ 0.01), whereas it was negatively associated with Peer Connectedness (β = −0.18, *p* ≤ 0.05). School Absence was negatively associated with LH (β = −0.19, *p* ≤ 0.01).

Indirect associations between learning behavior patterns and both School Absence and School Achievement were examined through mediation analyses (see Table 3). The results supported the hypotheses mediating the roles of Academic Procrastination and Affirming Diversity in the relationship between learning behavior patterns and School Achievement. Specifically, LH was indirectly associated with lower levels of School Achievement through Academic Procrastination and Affirming Diversity (β = −0.01, *p* ≤ 0.05), whereas MO was indirectly associated with higher levels of School Achievement through Academic Procrastination and Affirming Diversity (β = 0.02, *p* ≤ 0.05).

## 5. Discussion

School success—defined by school achievement and attendance—is related to individual factors (e.g., learning behavior patterns, procrastination) and contextual factors (e.g., school climate). However, school achievement and attendance show partially distinct patterns and are discussed separately where relevant. This study examined the sequential mediating role of procrastination and school climate in the relationship between LH, MO, and school success. We hypothesized that LH was expected to be positively associated with procrastination and negatively associated with school climate and school success, whereas MO would be negatively associated with procrastination and positively associated with school success. In line with the literature (Daily et al., 2020; Aldridge & Ala’I, 2013; J. Kim & Gentle-Genitty, 2020; Filippello et al., 2020; Diener & Dweck, 1980; Bäulke et al., 2021; Klassen & Kuzucu, 2009; Lee, 2005), our results, partially confirming our hypotheses, show that MO, LH, procrastination, and school climate are significantly associated with students’ school experience, highlighting their relationships with both school achievement and school attendance. Specifically, as highlighted in recent studies (Shan & Selamat, 2025; Meiri & Alfarraj, 2025; Theobald et al., 2024, 2023; Howell & Watson, 2007; Scher & Osterman, 2002; Wolters, 2004), our results show a positive association between LH and procrastination and a negative association between MO and procrastination. These findings are consistent with previous research suggesting that students who perceive themselves as incompetent and fear failure may be more likely to avoid starting or completing academic tasks (Buzzai et al., 2021; Krihadi et al., 2018). Conversely, the negative association observed between MO and procrastination is consistent with prior evidence indicating that students with greater self-regulation skills tend to tackle activities and tasks immediately, committing themselves to completing them right away and avoiding procrastination (Krihadi et al., 2018). Furthermore, students with MO, attributing intrinsic value to learning, are highly motivated to achieve school success (Sorrenti et al., 2018; Poredos & Puklek, 2017). Our results show a positive association between MO and school achievement and a negative association with absences, highlighting how these students are highly motivated to engage in academic challenges, participate regularly in activities, and achieve higher academic performance. Furthermore, procrastination, which is often linked to a fear of failure, has been associated with increased worry about not meeting certain academic standards and with variations in students’ school experience (Huang et al., 2025; Theobald et al., 2023). In fact, as can be inferred from the literature, procrastination has been associated with different students’ school lives, impacting aspects such as peer relationships (Jin et al., 2019), teacher relationships (Wang et al., 2023; Codina et al., 2018) and attachment to the school environment (Çıkrıkçı & Erzen, 2020; Erzen & Çikrıkci, 2018). Our results highlight the negative association between procrastination and all aspects of the school climate. This may be because students who tend to put off educational activities or tasks often participate less in school life (Caraway et al., 2003). Procrastination has been associated with fears of failure, making mistakes, or appearing inadequate (González-Brignardello et al., 2023; Schwinger et al., 2009; Wolters, 2003; Schouwenburg, 1992). These fears may be associated with lower levels of involvement in interactions with teachers (Teacher Support) and peers (Peer Connectedness), as well as with a reduced sense of school belonging (School Connectedness) and weaker perceptions of affirmation of individuality (Affirming Diversity). This could also alter their perception of school organization, associated with a lower tendency to use the services offered by the school. In this sense, they may consider the clarity of the rules (Rule Clarity) to be less adequate and perceive the institution as less willing to allow the reporting of abuse or violations and to provide support (Reporting and Seeking Help). Furthermore, a positive school climate is crucial for school success (La Rocca et al., 2024; Sorrenti et al., 2024; Daily et al., 2020; J. Kim & Gentle-Genitty, 2020; Credé et al., 2010). Our results show that connections with peers and the school, as well as affirming diversity, are associated with school achievement. Specifically, a negative association emerged between Peer Connectedness and School Achievement, in line with previous studies suggesting that, during adolescence, students tend to prioritize social aspects over academic ones, and that the quality of peer relationships significantly influences learning processes and academic performance (de Carvalho et al., 2025; Krnjajić, 2002; Fuligni et al., 2001). This pattern can be further understood in light of research on peer influence processes, which show that adolescents tend to form friendships with peers exhibiting similar levels of school engagement and performance, including those with lower achievement or higher absenteeism, thereby potentially reinforcing existing behavioral patterns (Shen & French, 2024; Rambaran et al., 2017). Indeed, during adolescence, peer influence is often associated with a gradual shift in focus away from academic commitments towards social and relational dimensions, resulting in a reduction in the importance attributed to school attendance and activities. Consistently, the association with less school-oriented peers has been linked to greater academic difficulties and lower achievement (Jacobson & Burdsal, 2012).

At the same time, our findings show that a strong connection to school and the appreciation of one’s cultural and social identity are positively associated with School Achievement. A welcoming, inclusive school environment that views diversity as an asset has consistently been related to better performance among students (Sorrenti et al., 2025a; Kwong & Davis, 2015; Warner & Heindel, 2017). Regarding affirming diversity, our findings suggest an indirect association involving procrastination and this dimension of school climate in the relationship between LH, MO, and School Achievement. Indeed, analysis of indirect effects suggested that, via procrastination and affirming diversity, LH is negatively associated with School Achievement. Conversely, MO is positively associated with School Achievement via the same mediated pathway. Students who attribute their school success to external factors and their failures to internal causes, related to a perception of poor personal ability, may be more likely to procrastinate due to a fear of failure. Such procrastination, understood as a form of avoidance linked to the fear of failure, can be linked with a perception of lower personal competence and a lower sense of valorization in the school context (affirmation of diversity). This reduction in the perception of recognition and acceptance may be associated with lower school achievement. Students who, on the other hand, approach academic difficulties and challenges with a focus on mastery and greater self-regulation of their studies show lower levels of procrastination. Reduced procrastination, in turn, is related to the affirmation of diversity—understood as the perception that valuing one’s own individuality is associated with better academic performance.

Considering the indirect effects identified in this study, procrastination and School Climate represent only some of the factors involved in the complex processes underlying academic success. The results partially support the model, as only certain aspects of School Climate were found to have a significant impact on academic performance, while others did not. This suggests that components of School Climate may not contribute equally to students’ academic experiences and may play different roles in relation to outcomes.

## 6. Conclusions

Overall, our results highlight the close interconnection between LH, MO, procrastination, and perceived School Climate, suggesting that interventions targeting procrastination and fostering an inclusive and welcoming school climate may be promising areas for further research and educational practice aimed at promoting positive academic experiences.

In an educational context, these results are especially helpful because they encourage a better understanding of the connection between motivation, emotions, and contextual factors in students’ educational experiences. They emphasize the importance of considering multiple dimensions when examining school success.

## 7. Limitations and Practical Implications

This study has limitations that must be considered in future research. Primarily, the cross-sectional design does not allow for the establishment of causal relationships. For this reason, future research should build on these findings by examining these associations across multiple time points and educational contexts. Longitudinal and experimental designs are needed to clarify the causal direction of the observed relationships.

Further methodological limitations concern the item parceling strategy. Although this approach is supported by methodological recommendations, its use remains debated within the structural equation modeling literature because it may reduce the transparency of the original measurement properties and obscure potential multidimensionality. Therefore, the findings should be interpreted with appropriate caution, considering the ongoing methodological debate surrounding item parceling.

Another limitation concerns the tools used for data collection, namely self-report questionnaires. While these tools are adequate and appropriate, they may introduce potential variance due to the common method effect and response bias shared across the variables under study, as well as foster biases related to social desirability. This factor, typical in an adolescent sample, could lead students to answer questions in a distorted way, trying to provide a positive self-image. Future studies could include mixed data collection methods, combining self-reports with direct observation tools.

School achievement and absence were also assessed through self-reporting, which may have reduced their accuracy, even though the students had access to their official school records. To overcome this limitation, future studies could draw directly on the objective data contained in school records (grades, absences) provided by the schools.

Furthermore, a limitation of the present study concerns the sample, which consisted of a convenience sample of secondary school students from Southern Italy. Although this approach enabled us to examine the proposed associations within a relatively homogeneous sample, it limits the ecological validity and generalizability of the findings to inclusive educational settings. Therefore, future studies should extend this line of research to other educational levels and explicitly include students with special educational needs in order to determine whether the observed associations are replicated across more diverse student populations. Despite these limitations, this study offers a significant contribution to the literature, providing useful insights for implementing strategies aimed at addressing variables associated with academic success. Although some indirect effects are small, they can guide us in developing more targeted educational strategies to support students’ learning paths. In fact, in the field of education, these findings offer a valuable contribution to understanding how motivational patterns, self-regulatory processes, and students’ perceptions of the school environment may jointly contribute to academic experiences. This perspective could help to identify specific areas for prevention and intervention aimed at supporting students’ adaptive functioning. The findings highlight how LH, MO, procrastination, and perceived School Climate are interconnected, suggesting that interventions aimed at reducing procrastination and promoting an inclusive and supportive school climate can significantly contribute to students’ academic success and personal development. In particular, addressing procrastination can alleviate underlying emotional distress and break the vicious cycle of avoidance. Over time, this cycle fuels the accumulation of tasks, alters one’s perception of their school environment, and promotes an increase in negative emotions (Theobald et al., 2023). In line with recent literature, training interventions aimed at developing self-regulation skills (Naderi et al., 2021; Grunschel et al., 2018; Lukas & Berking, 2018), mindfulness interventions (Sorrenti et al., 2025b; Rad et al., 2023; Dionne, 2016) and interventions focused on resilient coping (Meduri et al., 2026), which strengthen awareness of one’s own functioning in an educational context, have proven particularly effective in reducing procrastination, improving academic performance, and increasing student satisfaction and happiness. Furthermore, interventions based on acceptance and mindfulness approaches have proven effective in reducing academic procrastination, helping students to better manage their school behavior and perception of the educational context (Grunschel et al., 2018). Overall, these training programs improve students’ ability to cope with academic challenges, reducing procrastination and promoting a more positive experience of school that supports success.

Furthermore, given that peer relationships appear to exert a significant influence on adolescents, sometimes even directing their behavior away from school achievement, it is important to highlight several educational implications regarding the need for structured interventions. School programs should harness and positively channel these relational dynamics through cooperative learning strategies, with the aim of promoting collaboration, motivation, engagement, and academic achievement. The literature indicates that cooperative learning is more effective than individualistic approaches in promoting academic performance, particularly when students have opportunities to interact with peers and receive support and guidance from teachers (Hsiung, 2012; Gull & Shehzad, 2015). Furthermore, cooperative learning is associated with greater intrinsic motivation, greater task persistence, and the use of higher-order cognitive strategies compared to competitive and individualistic contexts (Johnson & Johnson, 1986).

## Figures and Tables

**Table 1:** Descriptive statistics and internal consistency for study variables.

Variable	Mean	SD	Min	Max	Skewness	SE Skewness	Kurtosis	SE Kurtosis	Cronbach’s α
**School Achievement**	7.40	0.91	6	10	0.44	0.105	−0.35	0.210	–
**School Absence**	23.11	11.8	0	80	0.78	0.105	0.36	0.210	–
**Academic Procrastination**	2.72	1.15	1	5	0.31	0.105	−0.99	0.210	0.87
**Mastery Orientation**	3.48	0.79	1	5	−0.39	0.105	0.02	0.210	0.82
**Learned Helplessness**	2.94	1.00	1	5	−0.00	0.105	−0.79	0.210	0.78
**Teachers Support**	3.18	0.95	1	5	−0.06	0.105	−0.70	0.210	0.90
**Peer Connectedness**	3.65	0.94	1	5	−0.66	0.105	−0.12	0.210	0.86
**School Connectedness**	3.35	0.98	1	5	−0.35	0.105	−0.55	0.210	0.88
**Affirming Diversity**	3.80	0.93	1	5	−0.73	0.105	0.03	0.210	0.89
**Rule Clarity**	3.66	0.90	1	5	−0.46	0.105	−0.12	0.210	0.90
**Reporting and Seeking Help**	3.58	0.99	1	5	−0.42	0.105	−0.36	0.210	0.91

***Note*:** N = 539.

**Table 2:** Correlational analyses for study variables.

	1		2		3		4		5		6		7		8		9		10		11
**1.** **School Achievement**	—																				
**2.** **School Absence**	−0.39	***	—																		
**3.** **Academic Procrastination**	−0.28	***	0.27	***	—																
**4.** **Mastery Orientation**	0.33	***	−0.17	***	−0.42	***	—														
**5.** **Learned Helplessness**	−0.10	*	0.04		0.29	***	−0.09	*	—												
**6.** **Teachers Support**	0.12	**	−0.10	*	−0.27	***	0.32	***	−0.13	**	—										
**7.** **Peer Connectedness**	0.08		−0.04		−0.11	*	0.32	***	−0.15	***	0.37	***	—								
**8.** **School Connectedness**	0.20	***	−0.11	**	−0.28	***	0.42	***	−0.20	***	0.60	***	0.70	***	—						
**9.** **Affirming Diversity**	0.23	***	−0.15	***	−0.12	**	0.38	***	0.04		0.39	***	0.43	***	0.51	***	—				
**10.** **Rule Clarity**	0.15	***	−0.12	**	−0.24	***	0.40	***	−0.03		0.51	***	0.37	***	0.59	***	0.57	***	—		
**11.** **Reporting and Seeking Help**	0.14	***	−0.08		−0.16	***	0.34	***	−0.06		0.56	***	0.42	***	0.59	***	0.51	***	0.67	***	—

***Note:*** N = 539; * *p* < 0.05, ** *p* < 0.01, and *** *p* < 0.001.

**Table 3:** Path estimates, SEs, and 95% CIs for tested direct, indirect, and covariational paths.

	*β*	*SE*	*Lower Bound (BC)* *95% CI*	*Upper Bound (BC)* *95% CI*	*p*
*Direct Effect*					
Learned Helplessness → Academic Procrastination	0.27	0.06	0.16	0.39	≤0.001
Mastery Orientation → Academic Procrastination	−0.50	0.09	−0.94	−0.59	≤0.001
Academic Procrastination → Teacher Support	−0.32	0.03	−0.24	−0.12	≤0.001
Academic Procrastination → Peer Connectedness	−0.15	0.05	−0.23	−0.04	≤0.01
Academic Procrastination → School Connectedness	−0.34	0.05	−0.39	−0.12	≤0.001
Academic Procrastination → Affirming Diversity	−0.16	0.04	−0.20	−0.04	≤0.01
Academic Procrastination → Rule Clarity	−0.29	0.04	−0.30	−0.14	≤0.001
Academic Procrastination → Reporting and Seeking Help	−0.20	0.05	−0.29	−0.09	≤0.001
Mastery Orientation → School Achievement	0.38	0.15	0.70	1.27	≤0.001
Peer Connectedness → School Achievement	−0.18	0.15	−0.65	−0.05	≤0.05
School Connectedness → School Achievement	0.22	0.20	0.03	0.84	≤0.05
Affirming Diversity → School Achievement	0.22	0.13	0.23	0.77	≤0.001
Mastery Orientation → School Absences	−0.19	1.04	−5.4	−1.3	≤0.01
*Indirect Effect via Academic Procrastination and Affirming Diversity*					
Learned Helplessness → School Achievement	−0.01	0.01	−0.03	−0.00	≤0.05
Mastery Orientation → School Achievement	0.02	0.02	0.01	0.09	≤0.05

Note: *β* represents standardized coefficients. Only statistically significant relations are reported.

## Data Availability

The datasets generated and/or analyzed during the current study are not publicly available due to privacy and ethical restrictions but are available from the University of Messina (Luana Sorrenti) upon reasonable request.
